# Introduction and Psychometric Validation of the Resilience and Strain Questionnaire (ResQ-Care)— A Scale on the Ratio of Informal Caregivers' Resilience and Stress Factors

**DOI:** 10.3389/fpsyt.2021.778633

**Published:** 2021-11-24

**Authors:** Alexandra Wuttke-Linnemann, Svenja Palm, Lea Scholz, Katharina Geschke, Andreas Fellgiebel

**Affiliations:** ^1^Center for Mental Health in Old Age, Landeskrankenhaus (AöR), Mainz, Germany; ^2^Department of Psychiatry and Psychotherapy, University Medical Center Mainz, Mainz, Germany; ^3^Hospital for Psychiatry, Psychosomatic and Psychotherapy, Agaplesion Elisabethenstift, Darmstadt, Germany

**Keywords:** counseling, diagnostics, health, homeostasis, prevention, stress awareness

## Abstract

**Background:** Informal caregivers are a particularly vulnerable population at risk for adverse health outcomes. Likewise, there are many scales available assessing individual caregiver burden and stress. Recently, resilience in caregivers gained increasing interest and scales started to assess resilience factors as well. Drawing on a homeostatic model, we developed a scale assessing both caregivers' stress and resilience factors. We propose four scales, two covering stress and two covering resilience factors, in addition to a sociodemographic basic scale. Based on the stress:resilience ratio, the individual risk of adverse health outcomes and suggestions for interventions can be derived.

**Methods:** A total of 291 informal caregivers filled in the ResQ-Care as part of a survey study conducted during the second wave of the COVID-19 pandemic in Germany. Exploratory factor analysis was performed. Validity analyses were examined by correlations with the Brief Resilience Scale (BRS), the Perceived Stress Scale (PSS-4) and the Geriatric Depression Scale (GDS-15).

**Results:** The data fitted our proposed four-factor solution well, explaining 43.3% of the variance. Reliability of each scale was at least acceptable with Cronbach's α ≥0.67 and MacDonald's ω ≥0.68 for all scales. The two strain scales weighed more than the resilience scales and explained 65.6% of the variance. Convergent and discriminant validity was confirmed for the BRS and PSS-4, whereas the GDS-15 correlation pattern was counterintuitive.

**Conclusion:** The factor structure of the ResQ-Care scale was confirmed, with good indications of reliability and validity. Inconsistent correlations of the scales with the GDS-15 might be due to a reduced validity of GDS-15 assessment during the COVID-19 lockdown.

## Introduction

Informal caregivers are a vulnerable population often referred to as invisible, secondary patients (1). They provide the majority of care for home-dwelling care-dependent people (2). The activities they perform for the care-dependent person are unpaid, exhausting, and often go unrecognized. As the caregiving experience can lead to chronic stress, caregivers are at increased risk for the development of physical and mental health impairments. Current estimates suggest an increased risk of depression in family caregivers (odds ratio 1.54), with estimated prevalence rates of depression lying at 10.1% in caregivers compared to 6.4% in non-caregivers (3).

Whereas, acute stress is adaptive, chronic stress directly translates into negative health consequences (4). In this regard, allostatic load and homeostasis are central concepts that may help understand how stress endangers health. Allostatic load can be regarded as the cumulative burden of stress (4). Homeostasis refers to the bodily equilibrium that individuals seek as it allows optimal functioning (5). Allostatic load threatens homeostasis and leads to negative health outcomes (6). Chronic stress further induces activations in stress-sensitive systems of the body and may lead to an inability to recover from stress. Caregivers often present with elevated stress levels (7), thus making them susceptible to the development of secondary physical and psychological impairments. It is assumed that the negative effects of caregiver burden on health are mediated by elevated biopsychological stress (8).

At the same time, not all caregivers experience negative health consequences. Indeed, Tuithof et al. (9) found that caregiving *per se* was not associated with mental health impairments. Specific risk factors have been identified that render some caregivers more susceptible to negative health consequences than others, such as lack of social support and limited access to resources (9), as well as certain sociodemographic factors (10).

In sum, there are huge interindividual differences in informal caregivers. While research has historically focused predominantly on the negative consequences of caregiving, the focus has recently begun to shift toward factors that predict better coping. In this regard, resilience, as the ability to bounce back in the face of adversity (11), has gained increasing interest. Resilience in caregivers can be conceptualized as a multidimensional construct (12) most often as biopsychosocial concept encompassing stable biological factors, individual psychological factors, and interpersonal social factors (13). Regarding biological factors, female caregivers present with higher resilience than male caregivers (13). Psychological factors such as self-efficacy represent important predictors of the psychological dimension of resilience (14). The social dimension of resilience encompasses quantity and quality of social contacts as well as perceived social support (14). Particularly the social dimension is thought to have profound effect in predicting resilience with researchers adding an interpersonal dimension of resilience that specifically focuses on the interpersonal relationship between caregiver and care recipient (8, 14, 15). Although resilience is presumed to be associated with beneficial effects on health, findings are heterogeneous yet. For example, during the COVID-19 pandemic there are also findings that higher resilience was associated with higher levels of anxiety during pandemic-related lockdown (16), thus making it necessary to better understand which mechanisms are underlying resilience in caregivers.

Overall, caregivers are often overseen in the health-care system as they tend to set aside their needs, making it harder to raise their awareness of low-threshold prevention. In this regard, caregivers are often hesitant to use support services for themselves (17, 18). Most often, caregivers of patients with dementia in particular state that support for themselves is not yet necessary (17), they do not consider themselves being a caregiver (19), and they fear a label as being needy and not competent enough to take care of the situation themselves (20).

We therefore developed the Resilience and Strain Questionnaire for Caregivers (ResQ-Care), as preventive diagnostic tool which visualizes the ratio between stress and resilience factors and thus puts an emphasis on resources and challenges likewise. It is conceptualized for a counseling context to motivate caregivers to invest in their health on a preventive way as well and to identify those caregivers that are particularly vulnerable concerning adverse health effects of caregiving.

### The Construction of the Resilience and Strain Questionnaire for Caregivers (ResQ-Care)

The items chosen for the ResQ-Care were identified by means of a literature review that focused on identifying (a) predictors of high stress and high caregiver burden and (b) predictors of resilience. Additionally, we searched for published scales on caregiver burden in the German language and considered constructs underlying high-loading items. A detailed description and justification for each item can be found in the manual in which each item is introduced individually (21).

In the context of health counseling, the ResQ-Care aims at identifying those caregivers at risk of developing stress-related health complaints due to greater experiences of stress and reduced capacities of resilience. Concerning the stress framework, the ResQ-Care model is based on the aforementioned homeostatic model. Concerning the resilience framework, we follow the biopsychosocial model on resilience in caregivers (13), with a particular emphasis on the social dimension of resilience.

Keeping in mind the equilibrium as represented visually by a scale, we aimed at developing a questionnaire that assesses stress and resilience factors in caregivers equally, and allows for them to be related to each other by means of a ratio.

We set out to explore the validity of the ResQ-Care by conducting a survey study in which we presented a total of 291 caregivers with the ResQ-Care in addition to items on resilience, depression, and stress, among others. We aim at performing exploratory factor analysis to examine underlying constructs of the ResQ-Care. Further, we set out to analyze convergent and discriminant validity by hypothesizing that the strain scales correlate positively with scores on depression and stress and negatively with resilience scores, whereas we hypothesize the opposite pattern concerning the resilience scales.

## Materials and Methods

### Study Sample

**Figure 1:**
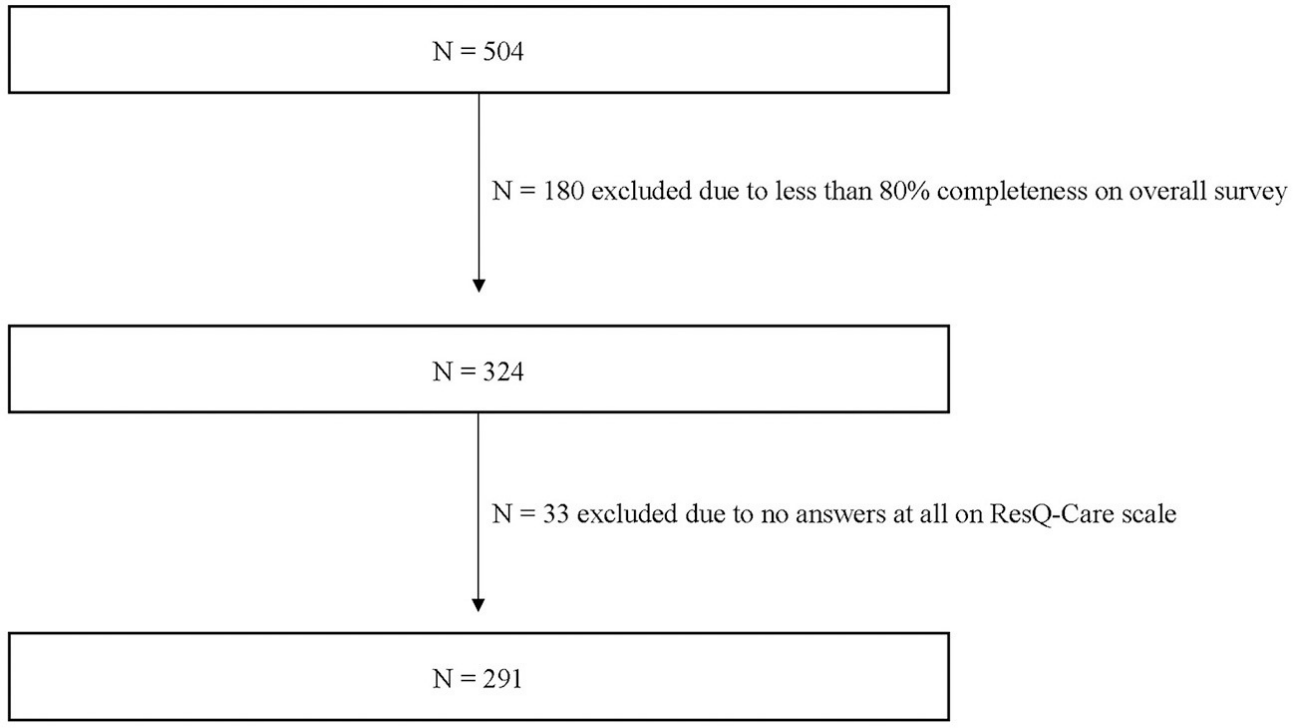
Flow chart of the study population selection.

The sample consists of *n* = 291 informal caregivers (see Figure 1). Caregivers were predominantly female (84.5%, *N* = 246) and on average 56.60 ± 10.63 years of age. Concerning the relationship to the care recipient, 51.4% (*N* = 149) of the participants were part of child-parent dyads with the child taking care of their parent, 33.8% (*N* = 98) were spousal caregivers, and 9.7% (*N* = 28) were parents taking care of their children.

Concerning characteristics of the care recipient, over half of the care recipients were female (59.1%, *N* = 172) and the mean age was 72.15 ± 19.61 years. Their level of care was rated at 3.23 ± 1.19 (levels of care range from 1 to 5 in Germany). At the time of study participation, recipients had already been care-dependent for an average of 7.49 ± 6.75 years.

A full review of sociodemographic characteristics can be found in Table 1.

**Table 1:** Sociodemographic characteristics of caregivers and care recipients (*n* = 291).

		**Caregivers**		**Care Recipient**	
		**%**	**X (±SD)**	**%**	**X (±SD)**
Gender	Female	84.5%		59.1%	
Age (in years)			56.60 (±10.63)		72.15(±19.61)
Employment	Part time or more	54.0%			
Income	>50,000 euros per annum	10.4%			
	30,000–50,000 euros per annum	22.6%			
	10,000–30,000 euros per annum	39.2%			
	<10,000 euros per annum	27.8%			
Educational qualification	Medium-track secondary school diploma or lower	46.4%			
Area of residence	Rural areas	51.9%			
	Towns or cities	48.1%			
Relationship to care recipient	Child taking care of parent	51.4%			
	Spousal caregiver	33.8%			
	Parents taking care of child	9.7%			
	Other	5.1%			
Hours spent caregiving before pandemic	<20 h/week	33.0%			
	20–40 h/week	33.0%			
	>40 h/week	34.0%			
					
Level of care (1–5)					3.23 (±1.19)
Physical impairment (0/1)				88.3%	
Dementia diagnosis (0/1)				47.6%	
Care dependent since (in years)					7.49 ± 6.75

*X, mean; SD, standard deviation, 0 = no, 1 = yes*.

### Study Procedure

The original German version of the ResQ-Care was administered (21). In German the scale is named “Fragebogen zur Angehörigenbelastung und Resilienz” (FARBE). For the purpose of this publication, the scale was translated into English by a professional translator leading to the title Resilience and Strain Questionnaire in Caregivers (ResQ-Care).

The original German scale was administered in paper-and-pencil format or online in addition to the BRS, GDS-15, and PSS-4, as part of an anonymous survey conducted between October 2020 and January 2021. During this time period for data acquisition, in October 2020 nationwide social restriction rules to contain the COVID-19 pandemic were active in Germany followed by a nationwide lockdown from November 2020 onwards. The survey was embedded in a larger study on caregivers' experience during the COVID-19 pandemic in Germany.

Recruitment for the online version of the survey was performed by distributing the link on various websites, whereas the printed version of the survey, along with the prepaid envelopes, was sent to registered physicians, outpatient clinics, care support offices, and welfare organizations, among others.

The study was approved by the ethics committee of the State Chamber of Physicians of Rhineland-Palatinate (Landesärztekammer RLP, processing number 2020-15286) and was pre-registered at the German Clinical Trials Register (DRKS; registration number DRKS00024621).

### The ResQ-Care

The ResQ-Care comprises a total of 20 items covering four scales in addition to a sociodemographic basis scale consisting of four items (Table 2).

**Table 2:** Overview of Scales assessed by ResQ-Care and X, SD, Min, and Max based on study sample.

		**Construct**	**X**	**SD**	**Min**	**Max**
Resilience scales	My strength-givers					
	My inner attitude	Psychological dimension of resilience	10.42	2.85	2	15
	My sources of energy	Social dimension of resilience	8.42	3.41	0	15
Strain scales	My strength-sappers					
	Difficulties in dealing with the person in need of care	Interpersonal dimension of caregiver burden	8.27	3.58	0	15
	General burdens of my living situation	Intrapersonal dimension of caregiver burden	8.81	3.81	0	15

*X, mean; SD, standard deviation, Min = minimum, Max = maximum*.

The sociodemographic basis scale is conceptualized as a screening scale that allows for the identification of caregivers who are at high risk of adverse health outcomes due to sociodemographic characteristics. These are based on a review by Adelman et al. (2), who identified, among other factors, that caregivers who were female, had low educational attainment (medium-track secondary school diploma or lower), spent many hours per week on caregiving tasks (>21 h/week), and cohabited with the care recipient were at greater risk of higher caregiver burden. The sum score of this scale ranges between 0 and 4 with higher figures indicating higher risk of caregiver burden.

The two resilience scales (“my strength-givers”) focus on the psychological dimension of resilience (“my inner attitude”, IA) and the social dimension of resilience (“my sources of energy”, SE). The items of the scale “my inner attitude” cover self-determination, self-efficacy, growth, and recovery from stress, among others. The items of the scale “my sources of energy” focus predominantly on social support and the scheduling of pleasant activities that exert an antidepressant effect.

The two strain scales (“my strength-sappers”) cover an interpersonal dimension (“difficulties in dealing with the person in need of care”, DIFF) and an intrapersonal dimension (“general burdens of my living situation”, GB) of caregiver burden. The scale “difficulties in dealing with the person in need of care” assesses primarily behavioral and psychological symptoms and impairments in activities of daily living concerning the care recipient. Additionally, dyadic aspects are covered by items on relationship quality. The scale “general burdens of my living situation” focuses on the caregiver him-/herself and comprises items on the caregiver's own health, role conflicts, and general stressors and burdens.

Each item is rated on a scale ranging from 0 (“no”), 1 (“rather no”), 2 (“rather yes”), to 3 (“yes”). The sum score for each scale is calculated and can vary between 0 and 15. All items are positively poled so that higher sum scores on the strain scales represent higher stress whereas higher sum scores on the resilience scales represent higher resilience.

The interpretation of the scale consists of two steps. Initially, the sociodemographic basis scale and the four scales are interpreted individually. For the sociodemographic basis scale, whenever a caregiver fulfills all four criteria, the cut-off values for the ensuing scales are lowered. For the two resilience scales, the cut-off is defined as atleast two items being answered with at least “rather no” or “no.” Concerning the two strain scales, the cut-off is defined as no less than two items being answered with at least “rather yes” or “yes,” respectively. Whenever the cut-off is reached, recommendations on counseling themes for the caregiver are provided (e.g., self-care, social support, psychoeducation, self-help groups). For all caregivers affirming all risk factors on the sociodemographic basis scale (sum score = 4), it is recommended to interpret individually all resilience and strain scales independently of whether the cut-off has been reached or not.

Second, the ratio between stress and resilience factors is interpreted. An automated tool is available to visualize the stress:resilience ratio. Based on this ratio, four types of caregivers with differing needs for support can emerge. However, please note that the interpretation of the stress:resilience ratio is so far based on a visual screening as the validation of the cut-offs is pending. In a next step, this ratio will be evaluated to account for the fact that when relating these values to each other more information is provided in comparison to interpretation of absolute values on strain and resilience scales separately.

(1) Caregivers with high stress and low resilience → particularly vulnerable and should be provided with support
(2) Caregivers with balanced stress and resilience (high stress, high resilience) → support on a preventive level on how to reduce stress should be provided
(3) Caregivers with balanced stress and resilience (low stress, low resilience) → support on a preventive level on how to increase resilience should be provided
(4) Caregivers with low stress and high resilience → no acute support necessary, except if caregivers wish for prevention.

The German version of the ResQ-Care scale (FARBE), a detailed user manual (21), and an automated interpretation tool are available online (https://www.zqp.de/frageboegen-farbe/) and can be used free of charge.

### Brief Resilience Scale (BRS)

To assess trait resilience, we used the German version of the Brief Resilience Scale (22). The BRS was created to assess resilience as the ability to bounce back or recover from stress. It includes six items, half of which are positively worded and the other half reverse-coded. Items are rated on a 5-point Likert scale (11), with higher mean scores representing higher resilience. The reliability and validity of the German adaptation of the BRS has been demonstrated by Chmitorz et al. (22). In our sample, Cronbach's Alpha for the BRS was high with α = 0.840. Additionally, we calculated McDonald's Omega, which led to a similar result of ω = 0.841.

### Perceived Stress Scale (PSS-4)

The PSS measures “the degree to which situations in one's life are appraised as stressful” (23). It is a widely used self-report instrument for depicting how participants experience situations as uncontrollable and overloading. The original 10-item scale has proven to be a reliable, valid, and economical instrument for assessing perceived stress (24), with acceptable psychometric qualities for the four-item short version. The PSS-4 consists of four items that are rated on a 5-point Likert scale ranging from 0 = “never” to 4 = “very often”. Higher sum scores represent higher perceived levels of stress. Cronbach's Alpha for PSS-4 measures in our sample was acceptable with α = 0.793, McDonald's Omega was ω = 0.801.

### Geriatric Depression Scale (GDS-15)

In the present study, we used the Geriatric Depression Scale in its short version (25). All 15 items have a simple dichotomous answer format (“yes” or “no”). Higher sum scores reflect higher levels of depression, with a cut-off of sum score ≥5 being representative of clinically relevant depressive symptoms. Internal consistency as measured by Cronbach's α was high with α = 0.851 and McDonald's Omega was similarly high with = 0.859 in our sample.

### Statistical Analyses

Data analysis was performed using JASP (Version 0.15). Missing values were dealt with by pairwise exclusion. To evaluate the structure of the ResQ-Care questionnaire with its four theoretically assumed scales, we performed exploratory factor analysis using maximum likelihood estimation. The number of factors retained was analyzed by parallel analysis. Following the recommendation by Costello and Osborne (26), we used oblique rotation (oblimin) in order to account for the fact that factors correlate with each other. Convergent and discriminant validity were calculated by means of regression analyses, correlating the four ResQ-Care subscales with GDS-15, PSS-4, and BRS scores. For reliability analysis, we report Cronbach's α and MacDonald's ω for all scales. Mean and standard deviation are presented where appropriate. *P* ≤ 0.05 were considered as significant.

## Results

Concerning the sociodemographic basis scale, 24.6% of the caregivers fulfilled all four risk criteria (female, lower education, >20 h/week of care, cohabitation) and would thus be rated as particularly vulnerable due to sociodemographic characteristics. A further 34.0% fulfilled three criteria, 23.5% fulfilled two criteria, and 16.1% fulfilled one criterion. Only 1.8% fulfilled none of these criteria. Concerning the subsequent four ResQ-Care scales, the highest scores were on the resilience scale “my inner attitude”, followed by (in descending order) the strain scale “general burdens of my living situation”, the resilience scale “my sources of energy”, and the strain scale “difficulties in dealing with the person in need of care” (Table 2).

### Exploratory Factor Analysis

Results of both the Bartlett test (χ^2^ = 2200.565, df = 190, *p* = <0.001) and the Kaiser-Meyer-Olkin measure of sampling adequacy (KMO = 0.84) verified the adequacy of the analysis. Based on exploratory factor analysis with oblimin rotation, a four-factor solution was proposed explaining 43.3% of variance. This solution supports the designated four ResQ-Care scales for the most part (see Table 3), with few cross-loadings. Factor 1 corresponds to “general burdens of my living situation” (GB) and explains 16.7% of the variance. Factor 2 corresponds to “difficulties in dealing with the person in need of care” (DIFF) and explains 11.7% of the variance. Factor 3 represents “my sources of energy” (SE), making up 9.1% of the total variance in the data. The items in “inner attitude” (IA) load highest on Factor 4, which in turn explains 5.8% of the variance. The ResQ-Care scales were constructed to measure and relate factors of strains (GB and DIFF) and resilience (IA and SE). The explained variance amounts to 28.4% for the strain scales and 14.9% for the resilience scales. Cronbach's α as an indicator of reliability was acceptable for GB, DIFF and SE scales (>0.70) but questionable for the IA scale (=0.67). McDonald's ω was ≥0.68 for all four scales. Reliability analyses of the original allocation of items (5 items for each scale) let to better indicators of reliability concerning the two resilience scales, whereas the two strain scales presented higher reliability resulting from EFA. Only four of the 20 included items do not load highest on their originally designated scales. The item “The person in need of care is physically restricted and needs support to carry out activities of daily living (e.g., getting dressed, washing, movement, eating), which is difficult for me to provide” (DIFF) was assigned to Factor 1 (λ = 0.21), while its loading on Factor 2 (corresponding to the designated scale) is λ = 0.18. Another item from the same DIFF scale (“I can't leave the person in need of care alone for an hour”) loads highest on Factor 1 (λ = 0.34) instead of Factor 2. The item “Despite the increased demands, I manage to pursue my own interests (such as hobbies, sport)” (SE) clearly also loads highest in Factor 1 (λ = −0.53) instead of Factor 3. The fourth item now allocated to Factor 1 (λ = −0.47) instead of its designated scale is “I recover quickly from stress” from the IA scale. The item “I feel joy in my everyday life, e.g., when I take part in pleasant activities” (SE) posed most difficulty in clearly assigning it to one factor, as it loaded similarly high on Factors 1 (λ = −0.23) and 4 (λ = 0.23), additionally to its designated Factor 3. All of these items concerned showed a plausible loading pattern in accordance to the construction of the ResQ-Care, meaning that items originally allocated to the resilience scales showed positive loadings with resilience scales and negative ones with strain scales, and vice versa.

**Table 3:** Loading pattern and factor loadings of items concerning exploratory factor analysis.

**Designated Scale**	**Item (English)**	**Factor 1 GB**	**Factor 2 DIFF**	**Factor 3 SE**	**Factor 4 IA**
GB	Besides the caring tasks, I am burdened by other difficulties in everyday life (e.g., my own state of health, worries about other family members, reconciling caring-family-work)	**0.56**	0.06	0.12	0.05
	I suffer daily from physical complaints (e.g., pain, shortness of breath, unwanted weight change, heart palpitations, dizziness, musculoskeletal disorders)	**0.60**	0.06	−0.05	0.10
	I am worried about my financial situation	**0.61**	−0.02	−0.10	0.09
	I neglect my own health (e.g., missing medical check-ups, lack of sleep, unhealthy diet)	**0.73**	−0.06	−0.11	0.11
	I feel like I can't keep up with the multitude of demands in my everyday life (which can manifest, for example, in listlessness, sleep problems, joylessness, or irritability)	**0.80**	0.08	0.07	−0.15
DIFF	The person in need of care shows difficult behaviors, which are a burden to me (e.g., rejects help, shows aggressive behavior, sleep disturbances, lack of interest)	0.02	**0.82**	0.02	0.07
	The person in need of care has changed for the worse due to the illness (e.g., is more irritable, more negative, less compassionate, has mentally deteriorated)	0.02	**0.79**	0.01	−0.00
	In everyday life, lots of conflicts and arguments with the person in need of care arise	−0.02	**0.77**	−0.03	−0.07
	The person in need of care is physically restricted and needs support to carry out activities of daily living (e.g., getting dressed, washing, movement, eating), which is difficult for me to provide	**0.21**	0.18	−0.09	0.01
	I can't leave the person in need of care alone for an hour	**0.34**	0.19	−0.16	0.26
SE	Despite the increased demands, I manage to pursue my own interests (such as hobbies, sport)	**−0.53**	0.02	0.17	0.07
	I involve other people (e.g., family members, friend, professional carers, external care offers) in the care	0.06	0.04	**0.69**	−0.01
	I receive supportive feedback for my achievements as a carer	−0.05	−0.08	**0.71**	−0.01
	I have people I can always rely on	−0.09	0.07	**0.62**	0.09
	I feel joy in my everyday life, e.g., when I take part in pleasant activities	−0.23	−0.06	**0.23**	0.23
IA	I voluntarily and deliberately chose to take on the role of carer	0.07	−0.19	0.06	**0.27**
	Through the demands of caring, I am discovering new, positive sides of myself, of the person in need of care, and/or of our relationship with each other	0.08	−0.28	0.20	**0.37**
	I recover quickly from stress	**−0.47**	−0.03	−0.07	0.31
	I have engaged in gathering information about the illness of the person in need of care and about support services, and I feel competent in the care I provide	−0.01	0.00	0.09	**0.59**
	I am able to rely on my abilities in difficult situations	−0.13	−0.02	−0.00	**0.42**
Sum of squared loadings		3.18	2.23	1.73	1.10
% of variance		16.7	11.7	9.1	5.8
α (original allocation)		0.79	0.71	0.74	0.70
α (current allocation)		0.82	0.81	0.71	0.67
ω (original allocation)		0.80	0.73	0.74	0.68
ω (current allocation)		0.83	0.81	0.72	0.68

*Highest factor loadings per item are in bold. GB, General burdens of my living situation; DIFF, Difficulties in dealing with the person in need of care; SE, My sources of energy; IA, My inner attitude; Sum of squared loadings, variance explained by each rotated factor. Scale is available online (www.zqp.de/frageboegen-farbe)*.

### Convergent and Discriminant Validity

Correlations among ResQ-Care subscales and the BRS, PSS-4, and GDS-15 are presented in Table 4. In the analysis presented we used pairwise deletion; the same pattern of results emerged when using listwise deletion instead. Regarding the BRS and PSS-4, the results confirm the convergent and discriminant validity: The BRS as a measure of resilience correlated positively with the resilience scales and negatively with the strain scales. PSS-4 sum scores correlated negatively with the resilience scales and positively with the strain scales. The GDS-15 sum score, however, was positively correlated with the resilience scales and negatively correlated with the strain scales. Overall, the BRS and PSS-4 were negatively correlated (*r* = −0.521, *p* < 0.001) whereas the GDS-15 and BRS were positively correlated (*r* = 0.510, *p* < 0.001). The PSS and GDS-15 were negatively correlated (*r* = −0.690, *p* < 0.001).

**Table 4:** Correlations of designated ResQ-Care scales with BRS, PSS-4, GDS-15.

**Scales**	**BRS**	**PSS-4**	**GDS-15**
My inner attitude	0.520	−0.403	0.334
My sources of energy	0.367	−0.465	0.566
Difficulties in dealing with the person in need of care	−0.274	0.447	−0.427
General burdens of my living situation	−0.370	0.583	−0.610

*All p < 0.001. Analyses were done by pairwise deletion. BRS, Brief Resilience Scale; PSS-4, Perceived Stress Scale; GDS-15, Geriatric Depression Scale*.

## Discussion

### Summary of Results

Exploratory factor analysis confirmed the structure of the ResQ-Care scale with its four factors. Indicators of reliability were acceptable. Convergent and discriminant validity were confirmed concerning correlations with well-validated scales on resilience and perceived stress. Validity concerning depressive symptoms as measured by the GDS-15 was counter-intuitive and may be explained by the special lockdown circumstances under which data acquisition took place.

### Psychometric Properties of the ResQ-Care

Overall, the structure of the ResQ-Care, with its four assumed scales, was confirmed. Nevertheless, a limited number of items showed cross-loadings and challenge our allocation of items to the respective scales. We designed the ResQ-Care scale to cover five items per scale. This allocation of items has practical reasons as the visualization of the results leads to a scale representing the weight of resilience factors relative to the weight of stress factors. With each scale having the same weight in this visualization, the interpretation of the stress:resilience ratio has high face validity and is intuitive for the user. Especially in view of the fact that the application of the ResQ-Care scale is predestined for professional counseling contexts, it has to be easy and intuitive to use and interpret. Given that the cross-loadings always confirmed the pattern of allocation—items covering resilience scoring high on resilience and low on burden, and vice versa—for practical reasons, we argue that the allocation of items to each scale should be maintained.

Concerning convergent and discriminant validity with the BRS and PSS-4, the validity of the ResQ-Care scale was confirmed. The correlation pattern shows the expected loadings and in particular it demonstrated that the ResQ-Care scales explain additional variance in stress and resilience that are not captured by the BRS and PSS-4. As both the BSS and PSS-4 were designed for very broad populations, it is important to keep in mind that burden and stress factors in informal caregivers share specific components that are not captured by the very general scales. In line with this, Zhou et al. (27) also advocate for a specific definition of resilience in the context of caregiving.

The results of the GDS-15 regression analysis, however, appear counterintuitive at first glance (e.g., the higher the depression score, the higher the resilience and the lower the stress) but may well be explained by the special circumstances under which our study was conducted: Various items of the GDS-15 might describe normal and judicious reactions to a pandemic instead of depressive symptoms in elderly people. For instance, it includes several behavioral items such as “have you given up many activities and interests?” The present survey specifically asked for an evaluation that “corresponded to how you felt during the lockdown.” This framing may have resulted in the questions surveying impairment due to the lockdown rather than actual depressive symptoms. In our sample, for example, the GDS-15 correlated with the subscale “my inner attitude' and the BRS score among others. This correlation is primarily attributable to the fact that none of the participants with a high GDS score considered themselves to be not resilient. Since this distribution pattern seems counterintuitive, it could indicate a selection bias: Individuals who felt very burdened by the COVID-19 lockdown and who were also not very resilient may have been less likely to participate in the study. A high level of stress with a strong “inner attitude” could indeed indicate resilience in this respect: Initial COVID-19 studies indicate that a positive appraisal style in particular might constitute a resilience factor during the pandemic (28). To enable a clear assessment of validity with regard to depressiveness, it would be important to re-administer the questionnaires outside of COVID-19 lockdown episodes.

### Strain Scales Have a Higher Weighting Than Resilience Scales

The finding that the strain scales explained more variance than the resilience scales is highly important, but also unsurprising given that well-validated predictors of caregiver burden cover precisely those factors with a strong negative impact [e.g., cognitive impairments of care recipient, behavioral symptoms, depressive symptoms of caregiver (2, 29–32)]. Furthermore, research has only just begun to examine the role of resilience and other protective factors in understanding caregiver burden. Thus, it will be necessary to further examine which factors best buffer the negative consequences of well-known factors contributing to caregiver burden. A further explanation for this pattern of findings may lie in the well-established negativity bias (33), which refers to people's tendency to better remember “negative” events and also to weigh them more heavily. In retrospective surveys, this might lead to the stressful factors playing a greater role for respondents than the protective factors. Especially in the context of counseling where individuals are seeking help due to stress “the negative” might generally outweigh possible protective factors. At the same time, this negativity bias revers into a positivity bias in older age with older adults showing the capacity for emotion regulation reasons to shift their attention to positive stimuli and remember these better in comparison to younger adults (34). As in this study the major part of caregivers were children taking care of their parents (middle aged adults), it might be an intriguing future research question whether these caregivers differ in their stress:resilience ratio from spousal caregivers (most often older adults).

### Limitations

Although the sample size was adequate to perform EFA, it was not adequate to additionally perform a confirmatory factor analysis or analyses of measurement invariance. Therefore, the results presented here provide first evidence for psychometric good properties of the German version of the ResQ-Care while at the same time future studies are needed to corroborate these findings in larger samples. Additionally, the allocation of items has to be discussed critically as we decided for practical reasons to keep the original allocation although some items scored higher on other factors. However, for feasibility reasons we consider it very important that each scale consists of the same number of items, so that burden and resilience scales can directly be weighed against each other. Concerning the sample size, it has also to be critically discussed that our sample was predominantly female, thus limiting the generalizability to male caregivers. Nevertheless, as the majority of informal caregivers are female (35), the study sample does appear to be representative of the gender distribution of caregiver samples. Furthermore, when interpreting the results, it has to be kept in mind that the survey was conducted during the second wave of the COVID-19 pandemic in Germany and the corresponding lockdown policy. As the pandemic has been associated with increased burden for informal caregivers (36), the responses to each item might be biased by these particular circumstances in which caregivers found themselves. At the same time, this might also have led to a selection bias, with only caregivers who felt well enough to fill in the questionnaire choosing to take part in the survey. Moreover, we conducted the study in German and thus the psychometric properties relate to the original German version. Future studies are necessary to validate the ResQ-Care in different settings, more heterogeneous caregiver subpopulations and also in English language. Nevertheless, the results presented here are encouraging, as they provide confirming evidence for the structure and psychometric qualities of the German version of the scale.

## Conclusion and Outlook

The ResQ-Care was developed to identify those caregivers at increased risk of adverse health outcomes. A one-size-fits-all approach for caregivers is not appropriate. Rather, it is necessary to take a balanced view on factors that increase stress and burden as well as those factors that increase resilience and may buffer the negative effects of stress. In light of this first evidence for good psychometric properties of the ResQ-Care, the next step will be to evaluate the implementation and feasibility of the scale in a professional counseling context. In this regard it is of utmost importance to validate the proposed cut-off values for each scale and to establish cut-off values for the stress:resilience ratio to ultimately define caregivers at adverse health risk.

## Data Availability Statement

The raw data supporting the conclusions of this article will be made available by the authors, without undue reservation.

## Ethics Statement

The study was approved by the Ethics Committee of the State Chamber of Physicians of Rhineland-Palatinate (Landesärztekammer RLP, processing number 2020-15286). Written informed consent for participation in this anonymous survey study was not required in accordance with the national legislation and the institutional requirements.

## Author Contributions

KG, AF, AW-L, and SP contributed to conception and design of the study. KG and LS organized data acquisition and the database. AW-L and LS performed the statistical analyses. AW-L wrote the first draft of the manuscript. SP, LS, KG, and AF wrote sections of the manuscript. All authors contributed to manuscript revision, read, and approved the submitted version.

## Conflict of Interest

The authors declare that the research was conducted in the absence of any commercial or financial relationships that could be construed as a potential conflict of interest.

## Publisher's Note

All claims expressed in this article are solely those of the authors and do not necessarily represent those of their affiliated organizations, or those of the publisher, the editors and the reviewers. Any product that may be evaluated in this article, or claim that may be made by its manufacturer, is not guaranteed or endorsed by the publisher.
